# (*E*)-3-(2-{2-[1-(3-Hy­droxy­phen­yl)ethyl­idene]hydrazin­yl}-1,3-thia­zol-4-yl)-2*H*-chromen-2-one

**DOI:** 10.1107/S1600536811012189

**Published:** 2011-04-07

**Authors:** Afsheen Arshad, Hasnah Osman, Chan Kit Lam, Madhukar Hemamalini, Hoong-Kun Fun

**Affiliations:** a School of Chemical Sciences, Universiti Sains Malaysia, 11800 USM, Penang, Malaysia; b School of Pharmaceutical Sciences, Universiti Sains Malaysia, 11800 USM, Penang, Malaysia; c X-ray Crystallography Unit, School of Physics, Universiti Sains Malaysia, 11800 USM, Penang, Malaysia

## Abstract

In the title compound, C_20_H_15_N_3_O_3_S, the thia­zole ring is approximately planar, with a maximum deviation of 0.003 (1) Å, and makes dihedral angles of 7.44 (6) and 1.88 (6)° with the hy­droxy-substituted phenyl ring and the pyran ring, respectively. The hydroxyl group is disordered over two sets of sites, with an occupancy ratio of 0.567 (3):0.433 (3). In the crystal, the major disorder component mol­ecules are connected *via* bifurcated (three-centre) O—H⋯O and C—H⋯O hydrogen bonds, generating *R* ^1^_2_(6) motifs and resulting in supra­molecular chains along the *a* axis. In the minor occupancy component, however, mol­ecules are connected *via* C—H⋯O hydrogen bonds, forming supra­molecular chains along the *b* axis. Furthermore, the crystal structure is stabilized by π–π inter­actions between the thia­zole rings [centroid–centroid distance = 3.5476 (7) Å].

## Related literature

For details of coumarin derivatives, see: Raghu *et al.* (2009 ▶); Gursoy & Karali (2003 ▶); Chimenti *et al.* (2010 ▶); Kamal *et al.* (2009 ▶); Kalkhambkar *et al.* (2007 ▶). For graph-set notation, see: Bernstein *et al.* (1995 ▶). For the stability of the temperature controller used in the data collection, see: Cosier & Glazer (1986 ▶). For the synthesis of (*E*)-2-(1-(3-hy­droxy­phen­yl)ethyl­idene)hydrazinecarbothio­amide, see: Greenbaum *et al.* (2004 ▶) and for that of 3-[ω-bromo­acetyl coumarin, see: Nadeem *et al.* (2009 ▶).
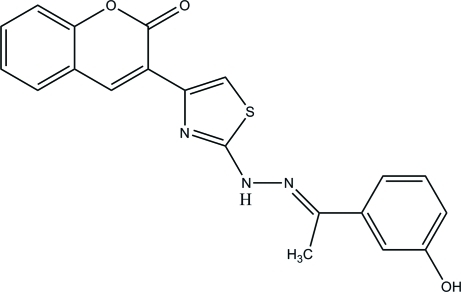


## Experimental

### 

#### Crystal data

C_20_H_15_N_3_O_3_S
*M* *_r_* = 377.41
Monoclinic, 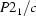

*a* = 9.1569 (1) Å
*b* = 9.9070 (2) Å
*c* = 18.7478 (3) Å
β = 92.040 (1)°
*V* = 1699.67 (5) Å^3^
*Z* = 4
Mo *K*α radiation
μ = 0.22 mm^−1^
*T* = 100 K
0.34 × 0.32 × 0.10 mm

#### Data collection

Bruker SMART APEXII CCD area-detector diffractometer
Absorption correction: multi-scan (*SADABS*; Bruker, 2009) ▶ *T*_min_ = 0.929, *T*_max_ = 0.979
29914 measured reflections
5400 independent reflections
4641 reflections with *I* > 2σ(*I*)
*R*_int_ = 0.030

#### Refinement

*R*[*F* ^2^ > 2σ(*F* ^2^)] = 0.041
*wR*(*F* ^2^) = 0.105
*S* = 1.07
5400 reflections
271 parameters
H atoms treated by a mixture of independent and constrained refinement
Δρ_max_ = 0.39 e Å^−3^
Δρ_min_ = −0.30 e Å^−3^

### 

Data collection: *APEX2* (Bruker, 2009 ▶); cell refinement: *SAINT* (Bruker, 2009 ▶); data reduction: *SAINT*; program(s) used to solve structure: *SHELXTL* (Sheldrick, 2008 ▶); program(s) used to refine structure: *SHELXTL*; molecular graphics: *SHELXTL*; software used to prepare material for publication: *SHELXTL* and *PLATON* (Spek, 2009 ▶).

## Supplementary Material

Crystal structure: contains datablocks global, I. DOI: 10.1107/S1600536811012189/wn2428sup1.cif

Structure factors: contains datablocks I. DOI: 10.1107/S1600536811012189/wn2428Isup2.hkl

Additional supplementary materials:  crystallographic information; 3D view; checkCIF report

## Figures and Tables

**Table 1:** Hydrogen-bond geometry (Å, °)

*D*—H⋯*A*	*D*—H	H⋯*A*	*D*⋯*A*	*D*—H⋯*A*
O3—H1*OA*⋯O2^i^	0.89 (4)	1.89 (4)	2.7693 (19)	171 (4)
C19—H19*A*⋯O2^i^	0.93	2.59	3.3020 (17)	133

Symmetry code: (i) 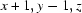.

**Fig. 1.**
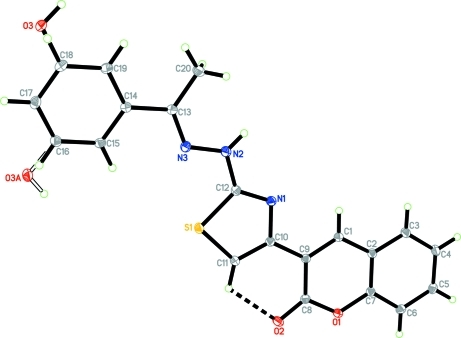
The asymmetric unit of the title compound, showing 30% probability displacement ellipsoids. The open bonds represents the minor disordered components.

**Fig. 2.**
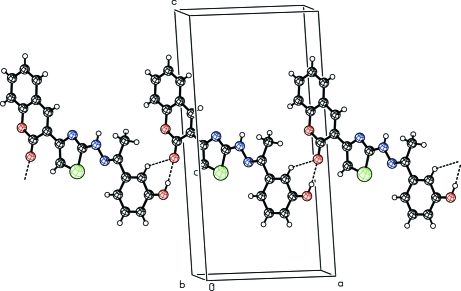
The crystal packing of the title compound, involving the major disorder components of the molecules. Dashed lines indicate hydrogen bonds.

**Fig. 3.**
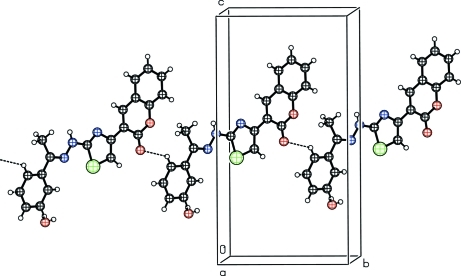
The crystal packing of the title compound, involving the minor disorder components of the molecules. Dashed lines indicate hydrogen bonds.

## supplementary crystallographic information

### Comment

Coumarin derivatives containing the thiazolyl unit exhibit promising antimicrobial activities against different microbial strains (Raghu *et al.*, 2009), including *Mycobacterium tuberculosis* (Gursoy *et al.*, 2003) and *Helicobacter pylori* (Chimenti *et al.*, 2010). These types of compounds are also reported to be good anticancer (Kamal *et al.*, 2009), analgesic and anti-inflammatory agents (Kalkhambkar *et al.*, 2007). The title compound is a new derivative of coumarin with the thiazole ring. We present here its crystal structure.

In the molecular structure of the compound, (Fig.1), the thiazole (S1/N1/C10–C12) ring is approximately planar, with a maximum deviation of 0.003 (1) Å for atom C11. The central thiazole (S1/N1/C10–C12) ring makes dihedral angles of 7.44 (6)° and 1.88 (6)° with the hydroxyl- substituted phenyl (C14–C19) ring and the pyran (O1/C1,C2/C7–C9) ring, respectively.  The hydroxyl group is disordered over two sites, with an occupancy ratio 0.567 (3):0.433 (3).

In the crystal packing (Fig. 2), the major component molecules are connected via bifurcated O3—H1OA···O2 and C19—H19A···O2 hydrogen bonds, generating  *R*^1^_2_(6) motifs, (Bernstein *et al.*, 1995), resulting in supramolecular chains along the *a*-axis. In the minor component, however, molecules are connected via C19—H19A···O2 hydrogen bonds, forming one-dimensional supramolecular chains along the *b*-axis (Fig. 3). Furthermore, the crystal structure is stabilized by π···π interactions between the thiazole (S1/N1/C10–C12) rings [centroid-centroid distance = 3.5476 (7) Å; -x, -y, 1-z].

### Experimental

(*E*)-2-(1-(3-Hydroxyphenyl)ethylidene)hydrazinecarbothioamide (Greenbaum *et al.*, 2004) and 3-[ω-bromoacetyl coumarin] (Nadeem *et al.*, 2009) were synthesized as reported in the literature. The title compound was prepared by treating (*E*)-2-(1-(3-hydroxyphenyl)ethylidene)hydrazinecarbothioamide (2.5 mmol) with 3-ω-bromoacetylcoumarin (2.5 mmol) in a chloroform-ethanol (2:1) mixture. The reaction mixture was refluxed for 2–3 hours at 60°C to yield dense yellow precipitates. The precipitates were filtered and boiled with water containing sodium acetate. The title compound was recrystallized as golden crystals from ethanol:chloroform (3:1).

### Refinement

Atoms H1N2, H1OA, H1OB and H11 were located in a difference Fourier map and refined freely [N—H = 0.85 (2) Å; O—H = 0.80 (5) and 0.89 (4) Å; C—H = 0.965 (17) Å]. The remaining H atoms were positioned geometrically [C—H = 0.93 Å for aromatic C and C—H = 0.96 Å for methyl C] and were refined using a riding model, with *U*_iso_(H) =  k*U*_eq_(C), where k = 1.2 for aromatic C and 1.5 for methyl C. A rotating group model was applied to the methyl groups. The hydroxyl group is disordered over two sites, with an occupancy ratio 0.567 (3):0.433 (3).

### Crystal data

**Table:**

C_20_H_15_N_3_O_3_S	*F*(000) = 784
*M**_r_* = 377.41	*D*_x_ = 1.475 Mg m^−^^3^
Monoclinic, *P*2_1_/*c*	Mo *K*α radiation, λ = 0.71073 Å
Hall symbol: -P 2ybc	Cell parameters from 9987 reflections
*a* = 9.1569 (1) Å	θ = 3.0–30.8°
*b* = 9.9070 (2) Å	µ = 0.22 mm^−^^1^
*c* = 18.7478 (3) Å	*T* = 100 K
β = 92.040 (1)°	Plate, yellow
*V* = 1699.67 (5) Å^3^	0.34 × 0.32 × 0.10 mm
*Z* = 4	

### Data collection

**Table:**

Bruker SMART APEXII CCD area-detector diffractometer	5400 independent reflections
Radiation source: fine-focus sealed tube	4641 reflections with *I* > 2σ(*I*)
graphite	*R*_int_ = 0.030
φ and ω scans	θ_max_ = 31.0°, θ_min_ = 2.2°
Absorption correction: multi-scan (*SADABS*; Bruker, 2009)	*h* = −12→13
*T*_min_ = 0.929, *T*_max_ = 0.979	*k* = −13→14
29914 measured reflections	*l* = −27→27

### Refinement

**Table:**

Refinement on *F*^2^	Primary atom site location: structure-invariant direct methods
Least-squares matrix: full	Secondary atom site location: difference Fourier map
*R*[*F*^2^ > 2σ(*F*^2^)] = 0.041	Hydrogen site location: inferred from neighbouring sites
*wR*(*F*^2^) = 0.105	H atoms treated by a mixture of independent and constrained refinement
*S* = 1.07	*w* = 1/[σ^2^(*F*_o_^2^) + (0.0429*P*)^2^ + 0.8634*P*] where *P* = (*F*_o_^2^ + 2*F*_c_^2^)/3
5400 reflections	(Δ/σ)_max_ < 0.001
271 parameters	Δρ_max_ = 0.39 e Å^−^^3^
0 restraints	Δρ_min_ = −0.30 e Å^−^^3^

### Special details

**Table:**

Experimental. The crystal was placed in the cold stream of an Oxford Cryosystems Cobra open-flow nitrogen cryostat (Cosier & Glazer, 1986) operating at 100.0 (1) K.
Geometry. All s.u.'s (except the s.u. in the dihedral angle between two l.s. planes) are estimated using the full covariance matrix. The cell s.u.'s are taken into account individually in the estimation of s.u.'s in distances, angles and torsion angles; correlations between s.u.'s in cell parameters are only used when they are defined by crystal symmetry. An approximate (isotropic) treatment of cell s.u.'s is used for estimating s.u.'s involving l.s. planes.
Refinement. Refinement of F^2^ against ALL reflections. The weighted R-factor wR and goodness of fit S are based on F^2^, conventional R-factors R are based on F, with F set to zero for negative F^2^. The threshold expression of F^2^ > 2σ(F^2^) is used only for calculating R-factors(gt) etc. and is not relevant to the choice of reflections for refinement. R-factors based on F^2^ are statistically about twice as large as those based on F, and R- factors based on ALL data will be even larger.

### Fractional atomic coordinates and isotropic or equivalent isotropic displacement parameters (Å^2^)

**Table:**

	*x*	*y*	*z*	*U*_iso_*/*U*_eq_	Occ. (<1)
S1	0.17023 (3)	0.06980 (3)	0.402397 (16)	0.02037 (8)	
O1	−0.19100 (10)	0.47170 (9)	0.56457 (5)	0.01965 (18)	
O2	−0.14551 (11)	0.40120 (11)	0.45684 (5)	0.0273 (2)	
O3	0.75724 (18)	−0.54395 (18)	0.31831 (9)	0.0234 (5)	0.567 (3)
H1OA	0.797 (4)	−0.556 (4)	0.362 (2)	0.040 (10)*	0.567 (3)
O3A	0.4193 (3)	−0.2752 (3)	0.18640 (12)	0.0273 (7)	0.433 (3)
H1OB	0.363 (5)	−0.216 (5)	0.196 (2)	0.028 (11)*	0.433 (3)
N1	0.15618 (11)	0.10211 (11)	0.53942 (6)	0.0183 (2)	
N2	0.31472 (13)	−0.07177 (12)	0.50380 (6)	0.0227 (2)	
N3	0.36764 (12)	−0.13166 (12)	0.44404 (6)	0.0202 (2)	
C1	−0.00034 (13)	0.28351 (13)	0.62488 (7)	0.0184 (2)	
H1A	0.0626	0.2198	0.6456	0.022*	
C2	−0.07700 (13)	0.37365 (13)	0.67004 (7)	0.0184 (2)	
C3	−0.06263 (14)	0.37246 (14)	0.74487 (7)	0.0228 (3)	
H3A	−0.0011	0.3101	0.7677	0.027*	
C4	−0.13980 (14)	0.46385 (14)	0.78482 (7)	0.0231 (3)	
H4A	−0.1304	0.4625	0.8344	0.028*	
C5	−0.23196 (14)	0.55839 (14)	0.75060 (7)	0.0214 (2)	
H5A	−0.2831	0.6198	0.7778	0.026*	
C6	−0.24800 (14)	0.56170 (13)	0.67707 (7)	0.0203 (2)	
H6A	−0.3089	0.6248	0.6544	0.024*	
C7	−0.17091 (13)	0.46850 (13)	0.63785 (6)	0.0175 (2)	
C8	−0.11845 (13)	0.38669 (13)	0.52030 (7)	0.0187 (2)	
C9	−0.01694 (13)	0.28851 (12)	0.55271 (6)	0.0167 (2)	
C10	0.06365 (13)	0.19757 (12)	0.50645 (6)	0.0169 (2)	
C11	0.05871 (14)	0.19493 (13)	0.43358 (7)	0.0194 (2)	
C12	0.21703 (13)	0.03050 (13)	0.49064 (7)	0.0183 (2)	
C13	0.45893 (13)	−0.23030 (13)	0.45086 (7)	0.0186 (2)	
C14	0.50587 (13)	−0.28993 (12)	0.38266 (7)	0.0179 (2)	
C15	0.43761 (13)	−0.25098 (13)	0.31754 (7)	0.0189 (2)	
H15A	0.3632	−0.1871	0.3171	0.023*	
C16	0.48063 (14)	−0.30725 (13)	0.25395 (7)	0.0210 (2)	
H16A	0.4359	−0.2796	0.2111	0.025*	0.567 (3)
C17	0.58993 (15)	−0.40455 (14)	0.25351 (7)	0.0232 (3)	
H17A	0.6179	−0.4426	0.2108	0.028*	
C18	0.65664 (14)	−0.44400 (14)	0.31770 (8)	0.0232 (3)	
H18A	0.7292	−0.5097	0.3178	0.028*	0.433 (3)
C19	0.61671 (14)	−0.38669 (13)	0.38209 (7)	0.0210 (2)	
H19A	0.6639	−0.4129	0.4246	0.025*	
C20	0.51371 (16)	−0.28472 (15)	0.52150 (7)	0.0262 (3)	
H20A	0.5374	−0.2111	0.5531	0.039*	
H20B	0.5995	−0.3383	0.5148	0.039*	
H20C	0.4394	−0.3396	0.5418	0.039*	
H1N2	0.338 (2)	−0.090 (2)	0.5468 (11)	0.038 (5)*	
H11	0.0039 (18)	0.2534 (18)	0.4013 (9)	0.029 (4)*	

### Atomic displacement parameters (Å^2^)

**Table:**

	*U*^11^	*U*^22^	*U*^33^	*U*^12^	*U*^13^	*U*^23^
S1	0.02210 (15)	0.02380 (16)	0.01531 (14)	0.00190 (11)	0.00199 (11)	−0.00074 (11)
O1	0.0222 (4)	0.0196 (4)	0.0170 (4)	0.0039 (3)	−0.0006 (3)	0.0017 (3)
O2	0.0337 (5)	0.0307 (5)	0.0174 (4)	0.0105 (4)	−0.0017 (4)	0.0032 (4)
O3	0.0226 (8)	0.0261 (9)	0.0213 (9)	0.0086 (6)	0.0010 (6)	−0.0031 (7)
O3A	0.0396 (14)	0.0249 (13)	0.0179 (11)	0.0025 (10)	0.0077 (9)	−0.0001 (9)
N1	0.0189 (5)	0.0191 (5)	0.0171 (5)	0.0013 (4)	0.0016 (4)	0.0008 (4)
N2	0.0244 (5)	0.0262 (6)	0.0176 (5)	0.0070 (4)	0.0024 (4)	0.0001 (4)
N3	0.0191 (5)	0.0224 (5)	0.0192 (5)	0.0025 (4)	0.0032 (4)	−0.0006 (4)
C1	0.0187 (5)	0.0186 (6)	0.0178 (5)	0.0025 (4)	−0.0005 (4)	0.0002 (4)
C2	0.0178 (5)	0.0197 (6)	0.0176 (5)	0.0012 (4)	−0.0006 (4)	−0.0014 (4)
C3	0.0225 (6)	0.0271 (7)	0.0186 (6)	0.0057 (5)	−0.0026 (5)	−0.0014 (5)
C4	0.0223 (6)	0.0289 (7)	0.0179 (6)	0.0029 (5)	−0.0013 (5)	−0.0045 (5)
C5	0.0193 (6)	0.0213 (6)	0.0236 (6)	0.0009 (5)	0.0018 (5)	−0.0048 (5)
C6	0.0186 (5)	0.0182 (6)	0.0240 (6)	0.0015 (4)	0.0000 (5)	−0.0001 (5)
C7	0.0178 (5)	0.0179 (5)	0.0167 (5)	−0.0012 (4)	−0.0004 (4)	0.0001 (4)
C8	0.0195 (5)	0.0176 (5)	0.0190 (6)	0.0007 (4)	0.0010 (4)	0.0011 (4)
C9	0.0167 (5)	0.0159 (5)	0.0174 (5)	−0.0003 (4)	0.0000 (4)	0.0005 (4)
C10	0.0172 (5)	0.0166 (5)	0.0169 (5)	−0.0009 (4)	0.0011 (4)	0.0010 (4)
C11	0.0219 (6)	0.0200 (6)	0.0164 (5)	0.0011 (4)	0.0003 (4)	0.0002 (4)
C12	0.0177 (5)	0.0199 (6)	0.0174 (5)	−0.0007 (4)	0.0014 (4)	0.0009 (4)
C13	0.0169 (5)	0.0194 (6)	0.0197 (6)	−0.0001 (4)	0.0015 (4)	0.0021 (4)
C14	0.0162 (5)	0.0175 (5)	0.0202 (6)	−0.0017 (4)	0.0019 (4)	0.0018 (4)
C15	0.0188 (5)	0.0167 (5)	0.0213 (6)	0.0005 (4)	0.0021 (4)	0.0028 (4)
C16	0.0240 (6)	0.0191 (6)	0.0201 (6)	−0.0022 (5)	0.0024 (5)	0.0026 (5)
C17	0.0266 (6)	0.0200 (6)	0.0234 (6)	−0.0014 (5)	0.0071 (5)	−0.0015 (5)
C18	0.0208 (6)	0.0192 (6)	0.0297 (7)	0.0026 (5)	0.0037 (5)	−0.0004 (5)
C19	0.0183 (5)	0.0204 (6)	0.0244 (6)	0.0011 (4)	0.0002 (5)	0.0016 (5)
C20	0.0286 (7)	0.0286 (7)	0.0213 (6)	0.0041 (5)	−0.0002 (5)	0.0043 (5)

### Geometric parameters (Å, °)

**Table:**

S1—C11	1.7216 (13)	C5—C6	1.3815 (18)
S1—C12	1.7382 (13)	C5—H5A	0.9300
O1—C8	1.3706 (15)	C6—C7	1.3887 (17)
O1—C7	1.3799 (15)	C6—H6A	0.9300
O2—C8	1.2151 (15)	C8—C9	1.4626 (17)
O3—C18	1.352 (2)	C9—C10	1.4678 (17)
O3—H1OA	0.89 (4)	C10—C11	1.3655 (17)
O3A—C16	1.403 (3)	C11—H11	0.965 (17)
O3A—H1OB	0.80 (5)	C13—C14	1.4858 (17)
N1—C12	1.2987 (16)	C13—C20	1.4996 (18)
N1—C10	1.3990 (16)	C14—C19	1.3964 (17)
N2—C12	1.3682 (16)	C14—C15	1.4057 (17)
N2—N3	1.3713 (15)	C15—C16	1.3858 (18)
N2—H1N2	0.85 (2)	C15—H15A	0.9300
N3—C13	1.2894 (16)	C16—C17	1.3898 (18)
C1—C9	1.3570 (17)	C16—H16A	0.9300
C1—C2	1.4316 (17)	C17—C18	1.386 (2)
C1—H1A	0.9300	C17—H17A	0.9300
C2—C7	1.3964 (17)	C18—C19	1.3946 (19)
C2—C3	1.4044 (17)	C18—H18A	0.9300
C3—C4	1.3849 (18)	C19—H19A	0.9300
C3—H3A	0.9300	C20—H20A	0.9600
C4—C5	1.4009 (19)	C20—H20B	0.9600
C4—H4A	0.9300	C20—H20C	0.9600
			
C11—S1—C12	88.13 (6)	C10—C11—S1	110.77 (10)
C8—O1—C7	122.58 (10)	C10—C11—H11	127.8 (10)
C18—O3—H1OA	111 (2)	S1—C11—H11	121.4 (10)
C16—O3A—H1OB	102 (3)	N1—C12—N2	124.87 (12)
C12—N1—C10	109.05 (10)	N1—C12—S1	116.77 (10)
C12—N2—N3	114.89 (11)	N2—C12—S1	118.36 (9)
C12—N2—H1N2	118.2 (13)	N3—C13—C14	114.99 (11)
N3—N2—H1N2	126.9 (13)	N3—C13—C20	123.73 (12)
C13—N3—N2	119.58 (11)	C14—C13—C20	121.27 (11)
C9—C1—C2	121.80 (11)	C19—C14—C15	118.86 (12)
C9—C1—H1A	119.1	C19—C14—C13	120.81 (11)
C2—C1—H1A	119.1	C15—C14—C13	120.33 (11)
C7—C2—C3	118.15 (11)	C16—C15—C14	120.36 (12)
C7—C2—C1	118.13 (11)	C16—C15—H15A	119.8
C3—C2—C1	123.71 (12)	C14—C15—H15A	119.8
C4—C3—C2	120.22 (12)	C15—C16—C17	120.73 (12)
C4—C3—H3A	119.9	C15—C16—O3A	124.63 (15)
C2—C3—H3A	119.9	C17—C16—O3A	114.63 (15)
C3—C4—C5	120.00 (12)	C15—C16—H16A	119.6
C3—C4—H4A	120.0	C17—C16—H16A	119.6
C5—C4—H4A	120.0	O3A—C16—H16A	5.1
C6—C5—C4	120.91 (12)	C18—C17—C16	119.04 (12)
C6—C5—H5A	119.5	C18—C17—H17A	120.5
C4—C5—H5A	119.5	C16—C17—H17A	120.5
C5—C6—C7	118.34 (12)	O3—C18—C17	119.52 (14)
C5—C6—H6A	120.8	O3—C18—C19	119.37 (14)
C7—C6—H6A	120.8	C17—C18—C19	121.04 (12)
O1—C7—C6	117.37 (11)	O3—C18—H18A	2.7
O1—C7—C2	120.27 (11)	C17—C18—H18A	119.5
C6—C7—C2	122.37 (12)	C19—C18—H18A	119.5
O2—C8—O1	115.71 (11)	C18—C19—C14	119.94 (12)
O2—C8—C9	126.15 (12)	C18—C19—H19A	120.0
O1—C8—C9	118.14 (11)	C14—C19—H19A	120.0
C1—C9—C8	119.05 (11)	C13—C20—H20A	109.5
C1—C9—C10	121.73 (11)	C13—C20—H20B	109.5
C8—C9—C10	119.22 (11)	H20A—C20—H20B	109.5
C11—C10—N1	115.28 (11)	C13—C20—H20C	109.5
C11—C10—C9	127.12 (11)	H20A—C20—H20C	109.5
N1—C10—C9	117.59 (10)	H20B—C20—H20C	109.5
			
C12—N2—N3—C13	−179.10 (12)	C8—C9—C10—N1	−177.98 (11)
C9—C1—C2—C7	0.02 (19)	N1—C10—C11—S1	0.55 (14)
C9—C1—C2—C3	−179.70 (13)	C9—C10—C11—S1	−179.34 (10)
C7—C2—C3—C4	−0.2 (2)	C12—S1—C11—C10	−0.43 (10)
C1—C2—C3—C4	179.48 (13)	C10—N1—C12—N2	179.60 (12)
C2—C3—C4—C5	−0.4 (2)	C10—N1—C12—S1	0.00 (14)
C3—C4—C5—C6	0.3 (2)	N3—N2—C12—N1	−178.57 (12)
C4—C5—C6—C7	0.28 (19)	N3—N2—C12—S1	1.03 (15)
C8—O1—C7—C6	178.55 (11)	C11—S1—C12—N1	0.26 (11)
C8—O1—C7—C2	−1.91 (17)	C11—S1—C12—N2	−179.37 (11)
C5—C6—C7—O1	178.62 (11)	N2—N3—C13—C14	178.40 (11)
C5—C6—C7—C2	−0.91 (19)	N2—N3—C13—C20	−0.57 (19)
C3—C2—C7—O1	−178.63 (11)	N3—C13—C14—C19	172.60 (12)
C1—C2—C7—O1	1.64 (18)	C20—C13—C14—C19	−8.40 (18)
C3—C2—C7—C6	0.89 (19)	N3—C13—C14—C15	−8.30 (17)
C1—C2—C7—C6	−178.84 (12)	C20—C13—C14—C15	170.69 (12)
C7—O1—C8—O2	−179.37 (11)	C19—C14—C15—C16	−0.43 (18)
C7—O1—C8—C9	0.49 (17)	C13—C14—C15—C16	−179.54 (11)
C2—C1—C9—C8	−1.41 (18)	C14—C15—C16—C17	1.10 (19)
C2—C1—C9—C10	178.93 (11)	C14—C15—C16—O3A	179.92 (16)
O2—C8—C9—C1	−178.99 (13)	C15—C16—C17—C18	−0.56 (19)
O1—C8—C9—C1	1.17 (17)	O3A—C16—C17—C18	−179.49 (15)
O2—C8—C9—C10	0.7 (2)	C16—C17—C18—O3	176.29 (14)
O1—C8—C9—C10	−179.16 (11)	C16—C17—C18—C19	−0.7 (2)
C12—N1—C10—C11	−0.35 (15)	O3—C18—C19—C14	−175.63 (14)
C12—N1—C10—C9	179.55 (11)	C17—C18—C19—C14	1.3 (2)
C1—C9—C10—C11	−178.43 (13)	C15—C14—C19—C18	−0.76 (18)
C8—C9—C10—C11	1.90 (19)	C13—C14—C19—C18	178.35 (12)
C1—C9—C10—N1	1.68 (17)		

### Hydrogen-bond geometry (Å, °)

**Table:**

*D*—H···*A*	*D*—H	H···*A*	*D*···*A*	*D*—H···*A*
O3—H1OA···O2^i^	0.89 (4)	1.89 (4)	2.7693 (19)	171 (4)
C19—H19A···O2^i^	0.93	2.59	3.3020 (17)	133

Symmetry codes: (i) *x*+1, *y*−1, *z*.
